# Dual effect of aucubin on promoting VEGFR2 mediated angiogenesis and reducing RANKL-induced bone resorption

**DOI:** 10.1186/s13020-023-00786-w

**Published:** 2023-08-29

**Authors:** Yulin He, Hiotong Kam, Xue Wu, Qian Chen, Simon Ming Yuen Lee

**Affiliations:** 1 State Key Laboratory of Quality Research in Chinese Medicine and Institute of Chinese Medical Sciences, University of Macau, Avenida da Universidade, Taipa, 999078 Macao China; 2 Department of Pharmaceutical Sciences, Faculty of Health Sciences, University of Macau, Avenida da Universidade, Taipa, 999078 Macao China; 3 Center for Evolution and Conservation Biology, Southern Marine Science and Engineering Guangdong Laboratory (Guangzhou), Guangzhou, 511458 China; 4 Present Address: Department of Food Science and Nutrition, The Hong Kong Polytechnic University, Hung Hom, 999077 Hong Kong, China

**Keywords:** Aucubin, Pro-angiogenesis, Osteogenesis, Zebrafish, Endothelial cells, Medaka

## Abstract

**Background:**

Angiogenesis is regarded as a critical role in bone repair and regeneration, involving in pathological bone disorders such as osteoporosis. Aucubin, an iridoid glycoside primarily derived from *Eucommia ulmoides*, is reported to inhibit osteoclast activity, enhance bone formation and promote angiogenesis in osteoporosis models. Our study is to further investigate the anti-osteoporosis effect of aucubin in transgenic medaka, and the pro-angiogenic effect of aucubin and its mechanism of action both in vivo and in vitro.

**Methods:**

The anti-osteoporosis effect of aucubin was confirmed by using RANKL-stimulated bone resorption transgenic medaka. The pro-angiogenic effect of aucubin in vivo was investigated using vascular endothelial growth factor (VEGF) tyrosine kinase inhibitor II (VRI)-induced vascular insufficient transgenic zebrafish model. Furthermore, endothelial cell proliferation, migration, tube formation and the mechanisms were evaluated to identify the pro-angiogenic effect of aucubin in normal and su5416-injured human umbilical vein endothelial cells (HUVECs).

**Results:**

Aucubin decreased the resorption of the mineralized bone matrix and centra degradation in heat-shocked transgenic col10α1:nlGFP/rankl:HSE:CFP medaka. Moreover, aucubin reversed VRI-induced vascular insufficiency in zebrafish through regulating *flt1*, *kdr*, *kdrl*, *vegfaa*, *ang-1*, *ang-2*, *tie1* and *tie2* mRNA expressions in Tg(fli1a:EGFP)^y1^ or AB wild type zebrafish. Aucubin promoted cell proliferation by upregulating p-mTOR, p-Src, p-MEK, p-Erk1/2, p-Akt and p-FAK in HUVECs. Furthermore, aucubin exhibited a pro-angiogenic effect on su5416-injured HUVECs by promoting their proliferation, migration, and tube formation through regulating the phosphorylation of VEGFR2, MEK, ERK and the ratio of Bcl2-Bax.

**Conclusion:**

Aucubin could reduce bone resorption in RANKL-induced osteoporosis medaka by live imaging. Meanwhile, aucubin exhibited a protective effect in VRI-induced vascular insufficient zebrafish by regulating VEGF-VEGFR and Ang-Tie signaling pathways. Additionally, aucubin promoted the proliferation, migration and tube formation of HUVECs probably by mediating VEGFR2/MEK/ERK, Akt/mTOR and Src/FAK signalling pathways. This study further indicated the dual effect of aucubin on angiogenesis and osteogenesis which may be beneficial to its treatment of osteoporosis.

**Supplementary Information:**

The online version contains supplementary material available at 10.1186/s13020-023-00786-w

## Introduction

Osteoporosis is a metabolic bone disorder affecting millions of people worldwide, especially aged 50 years. [1, 2]. Bone is highly vascularized, in which the vascular system and angiogenesis are indispensable for bone development and regeneration by providing growth factors, O_2_, waste elimination, nutrients, hormones, neurotransmitters and renewable autologous cells [3–5]. Angiogenic endothelial cells (ECs), as a cellular highway for multiple types of cells, provide concerted interactions between bone homeostasis and metabolism [6]. Recently, it is reported that promoting angiogenesis and neovascularization in bone is a therapeutic option for the treatment of angiogenesis-dependent osteoporosis and osteogenesis [7, 8].

*Eucommia ulmoides*, as recorded in ancient books of Chinese medicine, was recommended for the treatment of osteoporosis for a long time [9]. Previous studies suggested that the anti-osteoporosis effects of *E. ulmoides* involved in angiogenesis, cell proliferation and inflammatory response via mediating various biological processes [10]. Aucubin, an iridoid glycoside primarily derived from *E. ulmoides*, displays potential effect to decelerate the development of osteoporosis through inhibiting osteoclast activity and enhancing bone formation in different osteoporosis models [11]. Recent studies showed that aucubin has the ability to promote the formation of vessels in ovariectomy mice and peripheral ischemia mouse [12, 13]. It suggests that angiogenesis may also be one of the mechanisms by which aucubin exerts its effect on osteogenesis in addition to promoting bone recovery.

In vivo imaging, one of the fastest growing fields for meeting the diverse research needs, offers the opportunity to directly define the detailed morphological size, location of organs and diseased tissue, cell shape changes and real-time observations [14]. Novel vertebrate models, including fish, have been used for skeletal and angiogenic research under both normal and pathological conditions. Due to their relative transparency, in vivo imaging of living specimens is allowed for noninvasive and the pathological changes in vivo are able to be observed clearly under imaging instruments [15]. The use of teleost fish such as medaka (*Oryzias latipes*) is increasing in popularity for analysis in skeletal research, especially for in vivo analysis of osteoblasts and osteoclasts [16, 17]. Zebrafish is a well-established in vivo model for studying angiogenesis by qualitatively and quantitatively analyzing the vascular phenotype, due to the high-throughput, genetic tractability, rapid development, low husbandry costs and convenience [18]. Additionally, transgenic technology improved in vivo imaging capabilities of zebrafish and medaka larvae [19, 20]. HUVECs are one of the most available and well-studied types of vascular endothelial cells, which have been widely used in angiogenesis-dependent bone formation studies [21, 22].

We attempted to investigate the involved mechanisms, in particular the effects of aucubin on angiogenesis by using HUVECs and transgenic zebrafish. In addition, the anti-osteoporosis effect of aucubin was further investigated by using RANKL-stimulated bone resorption transgenic medaka.

## Materials and methods

### Chemicals and reagents

Kaighn's modification of Han's F12 medium (F-12K), penicillin–streptomycin (PS), fetal bovine serum (FBS), phosphate buffered saline (PBS) and 0.25% (w/v) trypsin in ethylenediaminetetraacetic acid (EDTA) were purchased from Gibco (Maryland, USA). Dimethyl sulfoxide (DMSO), phosphatase inhibitor cocktail, heparin, paraformaldehyde (PFA), gelatin, tricaine, endothelial cell growth supplement and phenylmethylsulfonyl fluoride (PMSF) were purchased from Sigma-Aldrich Co. (St. Louis, MO, USA). Evo M-MLV RT Premix for qPCR Kit and SYBR Green Premix Pro Taq HS qPCR Kit were obtained from Accurate Biology (Shandong, China). VEGF was obtained from R&D Systems (Minneapolis, MN) and used as a positive control. VRI obtained from Calbiochem (Billerica, MA) was dissolved in DMSO. Aucubin was purchased from Chengdu MUST Bio-technology Co. (Sichuan, China). Su5416 and PD98059 were purchased from Sigma Sigma-Aldrich Co. (St. Louis, MO, USA). Radioimmunoprecipitation assay (RIPA) lysis buffer was purchased from Beyotime Biotechnology (Shanghai, China). All antibodies were purchased from Cell Signaling Technology (Danvers, MA, USA). The HRP-linked secondary antibody was purchased from Beyotime Biotechnology.

### Medaka maintenance and treatment

The effects of aucubin on RANKL-induced bone resorption were investigated using double transgenic col10α1: nlGFP/rankl:HSE:CFP medaka that allow in vivo imaging and express green fluorescent protein in osteoclasts to observe the origin and behavior of col10α1 positive cells during bone formation, degeneration and repair visually [23]. Col10α1 is one of the collagenous proteins of the bone matrix molecules that provide structural support to the bone. The double transgenic medaka could also be induced a strong osteoporosis-like phenotype by overexpressing RANKL after heat-shock treatment. It is characterised by the formation of ectopic osteoclasts, which severely degrade the new mineralised matrix in the spine and other skeletal structures [24]. Briefly, the transgenic medaka larvae at 9 days post fertilization (dpf) were used to induce RANKL expression by heat shock at 39 ℃ for 1.5 h. After heat shock and recover for 1 h at 30 ℃, larvae were then screened for successful RANKL induction, which express cyano-fluorescent protein (CFP) under a fluorescence microscope. In this study, larvae that have shown expression of both CFP and col10α1:nlGFP were selected and housed in six-well plates (eight larvae per well) to assess the effect of drug treatment. The larvae were immersed in 25 and 50 μM aucubin (dissolving in fish medium) for 5 days after heat shock.

### Live and fixed bone staining of medaka

Larvae were immersed into 0.1% Alizarin Complexone (ALC) dissolving in the fish medium as described for live staining [16]. 14 dpf larvae were incubated in the ALC staining solution for 2 h at 30 ℃ and then analyzed the staining under a fluorescence microscope using tetramethylrhodamine (TRITC) and GFP filter settings. Alizarin red staining was performed on fixed 14 dpf medaka larvae to investigate the bone mineralization in the axial skeleton as we have described [16].

### Maintenance of zebrafish and collection of embryos

Transgenic zebrafish Tg(*fli1a*-EGFP)^*y1*^ and AB wild-type zebrafish were obtained from the Institute of Chinese Medical Sciences (ICMS) and maintained as described previously [25]. Briefly, zebrafish were raised in a controlled environment (28.5 ℃ with a 14 h light/10 h dark cycle) and fed with brine shrimp twice daily. Embryos were collected from natural spawning within 30 min and cultured in embryo medium at 28.5 ℃. The embryonic membranes were carefully removed by repeated blowing using a plastic dropper at 24 h-post fertilization (hpf), and then distributed into a 24-well plate.

### Morphological observations of zebrafish embryos

Twenty-four hpf Tg (fli1a-EGFP) embryos were pretreated with 500 ng/mL VRI for 3 h. After VRI treatment, larvae were immersed in different concentrations of aucubin (3, 6, 12, 25 and 50 µM) or embryo water. After drug treatment for 24 h, the embryos were anesthetized with 1% (w/v) tricaine and observed for viability and morphological changes using Leica Fluorescence stereo microscopes.

### Cell line and culture

HUVECs were cultured in F-12 K nutrient mixture medium with 10% FBS, 100U/mL penicillin, 100U/mL streptomycin, 100 μg/mL heparin, 30 μg/mL endothelial cell growth supplement. 0.1% gelatin was coated on culture flasks, dishes and plates before use. Cells were grown at 37 ℃ in a humidified 5% CO_2_ atmosphere. All assays were done using low passage cells (3–8 passages).

### Growth curve assays using real-time cell analyzer (RTCA)

The RTCA system provide an advanced form of technology that makes it possible to monitor the growth of label-free cells in real-time, allowing for the estimate impedance changes in the culture medium [26]. A specially designed cell culture plate, E-plates, is incorporated to the cell culture wells. The cells are then attached to the cell culture wells and any changes in electronic impedance are recorded by sensors on the E-plates in the RTCA system. The change in electronic impedance is expressed as a cell index, a parameter indicating cell viability [27, 28]. The growth curves for different groups were performed in RTCA system and the effect of drug on cell proliferation along with time variation was analyzed continuously according to the previously described methods [29]. In brief, the RTCA system were incorporated to the E-plates to read and determine background impedance of the media first, ensuring that all wells of E-plates and the connections were functional before seeding the cells. Secondly, 5000 cells/well were inoculated in the E-plates. The RTCA connected E-plates were put into xCelligence instrument in a 5% CO_2_ incubator at 37 °C. Cell index was recorded automatically every 15 min intervals for 48 h.

### Cell viability assay

HUVECs were seeded into a 96-well plate (5 × 10^3^ cells per well) in F-12K complete media and cultivated for 24 h. The cells were treated with various concentrations (3–100 µM) of aucubin for 48 h. The cells of positive control group were received with VEGF (50 ng/mL). After 48 h incubation, cells were incubated in CCK8 for 2.5 h to measure the absorbance at 450 nm as reference wavelength using a FlexStation 3 Multi-Mode Microplate Reader (Molecular Devices, Sunnyvale, CA, USA). Moreover, to access the effect of aucubin on su5416-damaged HUVECs proliferation, the cell viability was also measured by CCK8 assay and RTCA system according to the manufacturer’s protocol. Briefly, the cells were treated with su5416 (2 μM) as model group. The drug groups were treated with different concentrations of aucubin (3.13, 6.25, 12.5, 25, or 50 μM) and su5416 (2 μM) for 48 h incubation. Cell viability data were calculated as the percentage of controls.

### Total RNA extraction, reverse transcription, and real-time quantitative PCR (qPCR)

Total RNA of HUVECs was extracted using the High Pure RNA Isolation Kit. Total RNA was isolated from zebrafish tissues using TRNzol reagent according to the manufacturer’s instructions. Isolated RNA was then reverse-transcribed into cDNA using the Evo M-MLV RT Premix for qPCR Kit (Accurate Biology, Shandong, China). The qPCR assay was conducted using SYBR Green Premix Pro Taq HS qPCR Kit (Accurate Biology, Shandong, China) with the QuantStudio 7 Flex Real-Time PCR System (ThermoFisher, USA). The amplification parameters were as follows: 50 °C for 2 min and 95 °C for 10 min, followed by 40 cycles of 95 °C for 15 s and 60 °C for 30 s. Each sample was analyzed in triplicate, and the relative expression of mRNA was calculated after normalization to elongation factor 1 alpha (elfα). The primer sequences used are listed in Table 1.

**Table 1:** Primers used for quantitative RT-PCR

Primer name	Sequence
*Flt-1* forward	5′-AACTCACAGACCAGTGAACAAGATC-3′
*Flt-1* reverse	5′-GCCCTGTAACGTGTGCACTAAA-3′
*kdr* forward	5′-CAAGTAACTCGTTTTCTCAACCTAAGC-3′
*kdr* reverse	5′- GGTCTGCTACACAACGCATTATAAC-3′
*kdrl* forward	5′-GACCATAAAACAAGTGAGGCAGAAG-3′
*kdrl* reverse	5′-CTCCTGGTTTGACAGAGCGATA-3′
*Ang-1* forward	5′-ACATGCAAGTGTGCACTGATGCTC-3′
*Ang-1* reverse	5′-TTCCGACATGCTGTCCTTGTCTGT-3′
*Ang-2* forward	5′-CCAATCTTCTAAGCCAATCAGCGGAA-3′
*Ang-2* reverse	5′-CCACATCTGTCAGTTTGCGCGTGTTT-3′
*Tie-1* forward	5′-AGAGGCACGGAAGGCTTATG-3′
*Tie-1* reverse	5′-TAGCCTCCCTTGGGCTATGA-3′
*Tie-2* forward	5′-TGAGCTACCTGAGCCAGAAACAGT-3′
*Tie-2* reverse	5′-TCTTCGCCACAAAGTTCTCTCCCA-3′
*Vegfaa* forward	5′-TGTAATGATGAGGCGCTCGAA-3′
*Vegfaa* reverse	5′-AGGCTCACAGTGGTTTTCTT-3′

### Wound healing assay

HUVECs were seeded into to 6-well plates. 200 μL micropipettor was used to generate scratches after the cells covered the bottom. In the model group, the cells were treated with su5416 (2 μM). Cells in the drug groups were treated with su5416 (2 μM) and 12.5, 25, or 50 μM aucubin. After 12 h incubation, the migratory impacts were observed and captured under a Leica DMi8 fluorescence microscope. The wound healing area was calculated by Image J software version 1.5.0.

### Tube formation assay

Matrigel Basement Membrane Matrix (BD, Biosciences) was thawed at 4 °C overnight and diluted with cell culture medium. 24-well plate was coated with Matrigel at 37 °C for 1 h for polymerization. 8 × 10^4^ HUVECs were seeded alone or resuspended in 500 µL medium containing different concentration of aucubin and su5416 on the Matrigel-coated plate. After 6 h incubation, control HUVECs formed tubular structures, defined as endothelial cords connected at both ends. HUVECs tubular-structures before and after the drug treatments were photographed using the Leica DMi8 fluorescence microscope. Tube formation was quantified by measuring tubular-structure length with Image J software version 1.5.0.

### Western blot analysis

Protein was extracted using RIPA lysis buffer containing 1% PMSF and 1% cocktail inhibitor after drug treatment. The protein content was determined using a BCA protein quantification kit (Thermofisher, Waltham, MA USA). Aliquots of protein samples (20–30 μg) were resolved by SDS-PAGE (7.5–15%) and then electrically transferred to 0.22 μm or 0.45 μm PVDF membranes. Membranes were subsequently blocked with 5% non-fat milk, followed by incubation with diluted primary antibodies (β-actin, VEGFR2, p-VEGFR2, mTOR, p-mTOR, Akt, p-Akt, Erk1/2, p-Erk1/2, MEK, p-MEK, Src, p-Src, FAK, p-FAK, Bcl-2 and Bax) at 4 °C overnight. The membranes were washed with PBST and incubated with horseradish peroxidase (HRP)-conjugated secondary antibodies for 1 h at room temperature. Proteins were visualized and scanned by enhanced chemiluminescence using a ChemiDoc XRS Imaging System (Bio-Rad, Hercules, CA, USA) following washes with PBST three times. The intensity of the protein bands was analyzed using Quantity One Software (Bio-Rad).

### Statistical analysis

All data are expressed as mean ± SD for at least three independent experiments. Data were analysed by GraphPad Prism 7.0. Statistical significance was assessed by One-way ANOVA analysis followed by post-hoc Dunnett’s test and post-hoc Tukey’s test, and a p-value less than 0.05 (p < 0.05) was considered significant.

## Results

### Aucubin treatments reduced RANKL-stimulated bone resorption in transgenic medaka

**Fig. 1:**
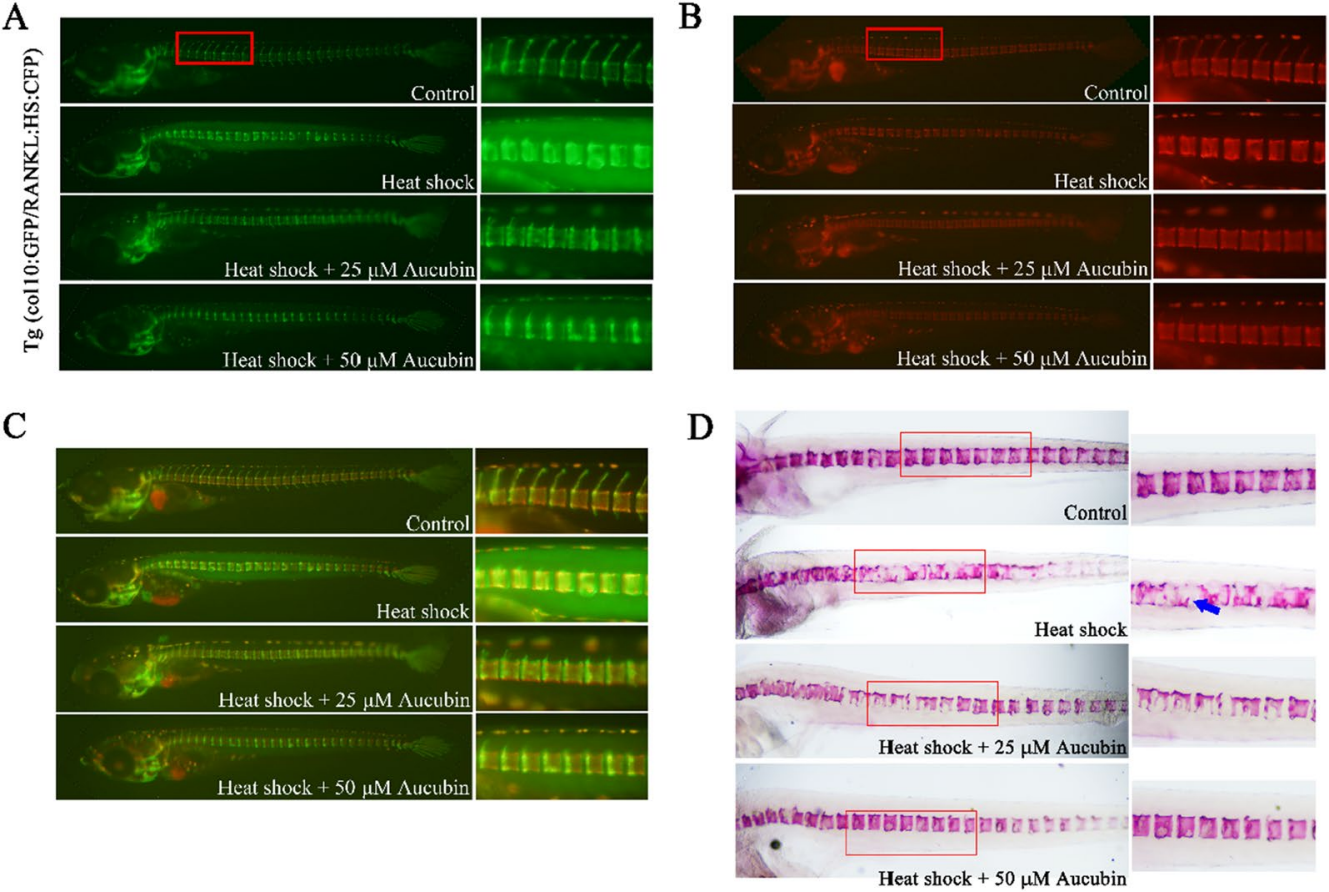
Aucubin improved bone recovery and reduced bone resorption in Col10α1: mGFP/ Rankl:HSE:CFP medaka. **A** Col10α1: nlGFP expression in 14 dpf transgenic medaka larvae after heat-shock-induced RANKL expression and aucubin (25 and 50 μM) treatments. **B** ALC staining of the mineralized bone matrix in 14 dpf Col10α1: mGFP/Rankl:HSE:CFP medaka, 5 days after heat-shock-induced RANKL expression and aucubin (25 and 50 μM) treatments. **C** A merge of **A** and **B**. **D** Fixed bone staining with Alizarin Red in 14 dpf Col10α1: mGFP/Rankl:HSE:CFP medaka larvae after RANKL induction and aucubin (25 and 50 μM) treatments

The efficacy of aucubin was assessed on RANKL-induced ectopic osteoclast formation in medaka model. Using 9 dpf transgenic col10α1:nlGFP/rankl:HSE:CFP medaka line, RANKL expression was induced by heat shock which increased the ectopic osteoclast formation. After heat shock, the medaka were immediately treated with 25 and 50 μM aucubin in the fish medium. No obvious induction of RANKL expression was observed in the control group and a mineralized vertebral column was continued to develop, along with normal neural and centra arches at 14 dpf (measured by fluorescence microscopy in Fig. 1A; live staining with Alizarin complexone (ALC) in Fig. 1B; staining with Alizarin red (AR) in Fig. 1D). In the RANKL-induced control fish, mineralized matrix resorption was significantly increased, resulting in loss of mineralized neural arches and lesions in the vertebral centra (Fig. 1A and B). However, 25 and 50 μM aucubin reduced RANKL-induced osteoclastogenesis (shown by fluorescence microscopy in Fig. 1A) and excessive bone resorption (shown by live ALC staining in Fig. 1B) by increasing the mineralized bone matrix. These results were also confirmed by AR staining of the medaka vertebral column (Fig. 1D).

### Aucubin treatments reduced VRI-induced vascular insufficiency in zebrafish

**Fig. 2:**
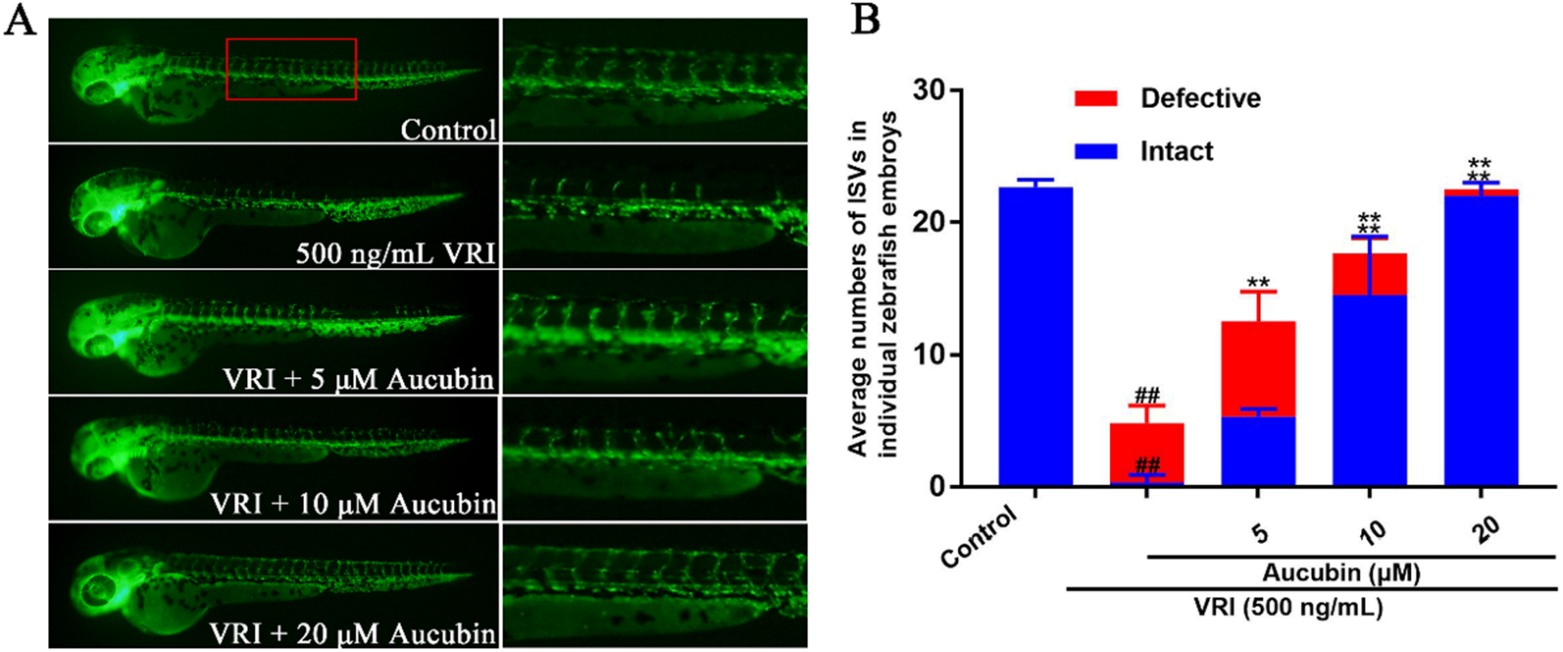
Aucubin reduced VRI-induced vascular insufficiency in zebrafish. **A** 24 hpf Tg(fli-1a:EGFP)^y1^ zebrafish embryos were pre-treated with 500 ng/mL VRI for 3 h, rinsed, and immersed in various concentrations of aucubin (5–20 µM) for 24 h. The expression of EGFP in ISVs of zebrafish embryos. **B** Quantitative analysis showing the concentration-dependent effects of aucubin on the recovery of ISVs. Data are presented as means ± SD for three independent trials. ^##^p < 0.01 versus the control group; **p < 0.01 versus the group treated with VRI alone

Transgenic zebrafish embryos, fli1a:EGFP, were used to evaluate the effect of aucubin on VRI-induced vascular insufficiency. As shown in Fig. 2A, 500 ng/mL VRI obviously inhibited the growth of intact intersegmental blood vessels (ISVs) in zebrafish embryos after pre-treatment for 3 h. However, after post-treatment with different concentrations of aucubin (5–20 μM), the number of intact and total ISVs was significantly elevated (Fig. 2A). Quantitative analysis confirmed a significant protective effect of aucubin on the recovery of blood vessel loss induced by VRI in zebrafish (Fig. 2B).

### Effects of aucubin on several pro-angiogenic factors mRNA expression in VRI-induced vascular insufficiency zebrafish

**Fig. 3:**
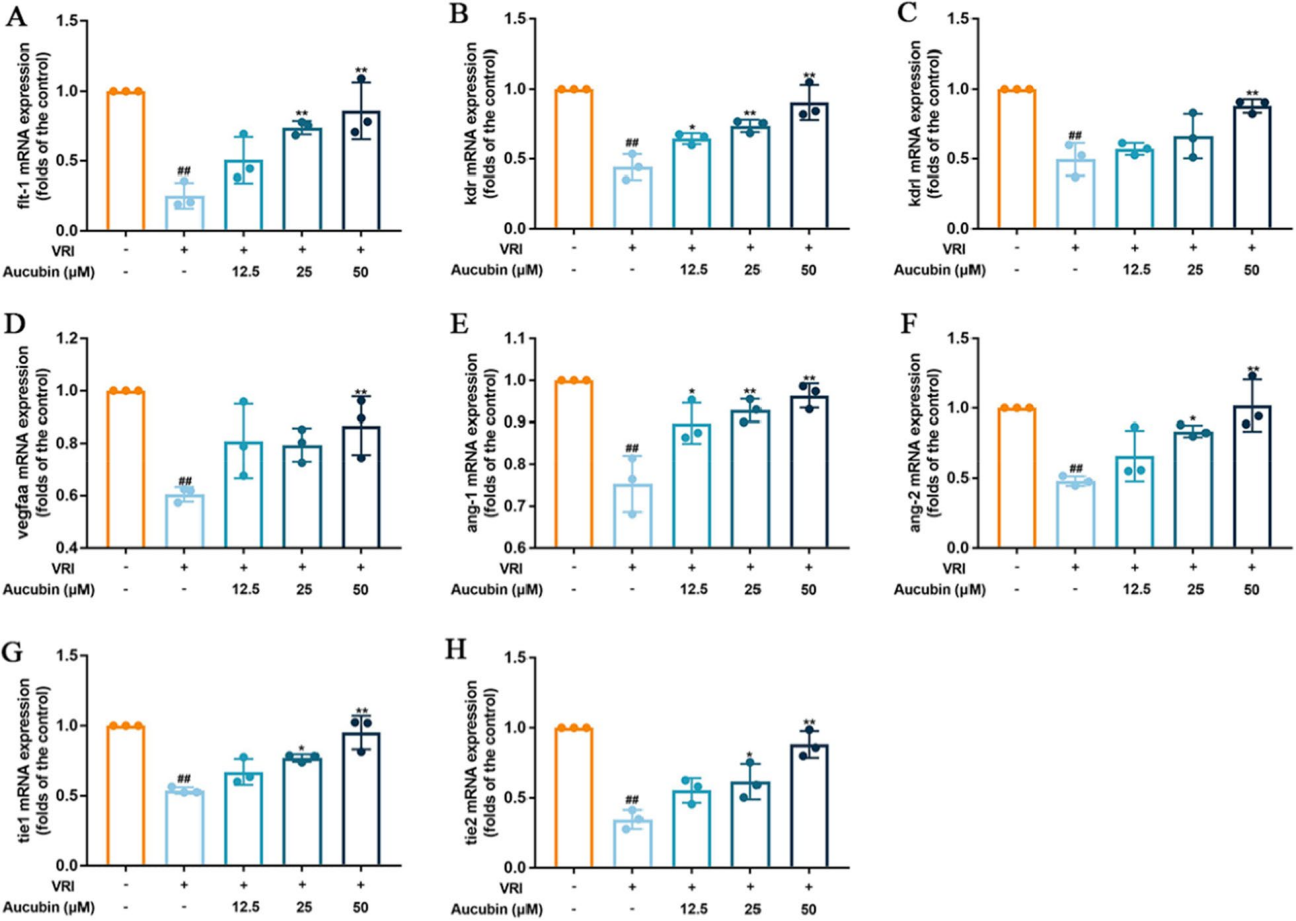
Effects of aucubin on VRI-induced down regulation of several pro-angiogenic factors mRNA expression in zebrafish. Total RNAs from different groups of zebrafish pre-treated with VRI (500 ng/mL) for 3 h, and then treated with different concentrations of Aucubin (5, 10 and 20 μM) after VRI withdrawal, were isolated and reverse transcribed to cDNA. The mRNA levels of flt-1 (**A**), kdr (**B**), kdrl (**C**), vegfaa (**D**), ang-1 (**E**), ang-2 (**F**), tie1 (**G**) and tie2 (**H**) were tested by RT-PCR. Data are expressed as the mean ± SD, ^##^p < 0.01 versus the control group; *p < 0.05 and **p < 0.01 versus the group treated with VRI alone

In order to confirm the vascular phenotypic effects of aucubin, the relative mRNA expressions of *flt-1*, *kdr*, *kdrl*, *ang-1*, *ang-2*, *vegfaa*, *tie1* and *tie2* were determined by real-time PCR. VRI induction caused a significant reduction in the above mRNA expressions. However, aucubin reversed the decreased expression of several key genes induced by VRI in zebrafish (Fig. 3A–H). These data provided a clue that aucubin exerted pro-angiogenic actions possibly via regulation of both VEGF-VEGFR (*flt1*, *kdr*, *kdrl* and *vegfaa*) and Ang-Tie (*ang-1*, *ang-2*, *tie1* and *tie2*) signaling pathways in zebrafish.

### Effects of aucubin on proliferation of HUVECs

**Fig. 4:**
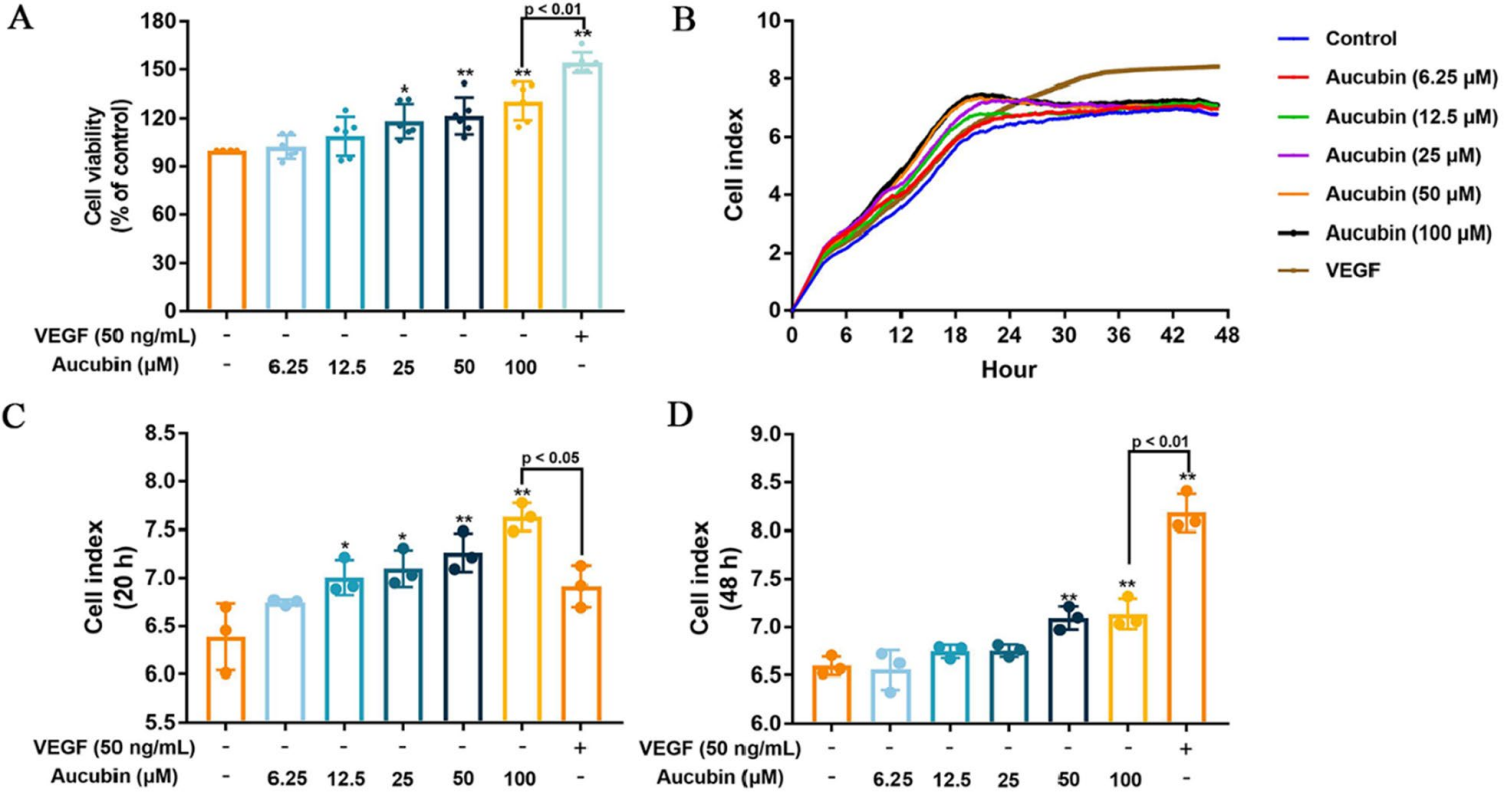
Effects of aucubin on proliferation in HUVECs. HUVECs were treated with different concentrations of aucubin (6.25–100 µM). **A** Cell viability was measured by CCK8 assays. **B** The xCELLigence Real-time cell analysis (RTCA) system showed the data of cell index curves of HUVECs reflected their proliferation in real-time mode. The cell index after aucubin administration at 20 h (**C**) and 48 h (**D**) were analyzed. Data are presented as the percentage of the control group (mean ± SD of three or six independent trials). **p < 0.01 versus the control group

To access the effect of aucubin on normal HUVECs proliferation, cell viability was evaluated by the CCK8 assay. Results showed that 25–100 μM aucubin increased the cell viability of HUVECs, and showed no cytotoxic effects within the concentration range of 6.25–100 µM (Fig. 4A). Moreover, these results were also confirmed by observing cell index for 48 h using xCELLigence Real-time cell analysis system (Fig. 4B). As shown in the Fig. 4B, cell index in the aucubin group increased more rapidly in comparison with the control and VEGF group during 0 h-20 h. Peak of cell index in aucubin group was reached resulting in a stable platform at 20 h, whereas VEGF group continued to increase during 20 h-48 h. Although both 50–100 μM aucubin and VEGF increased cell index at 48 h compared to control group (Fig. 4D), VEGF group showed significant difference compared to 100 μM aucubin group. These results demonstrated that aucubin promoted the proliferation of HUVECs without prolong effect causing excessive cell proliferation.

### Effects of aucubin on the expression and phosphorylation of key proteins involved in angiogenesis signaling pathway in HUVECs

**Fig. 5:**
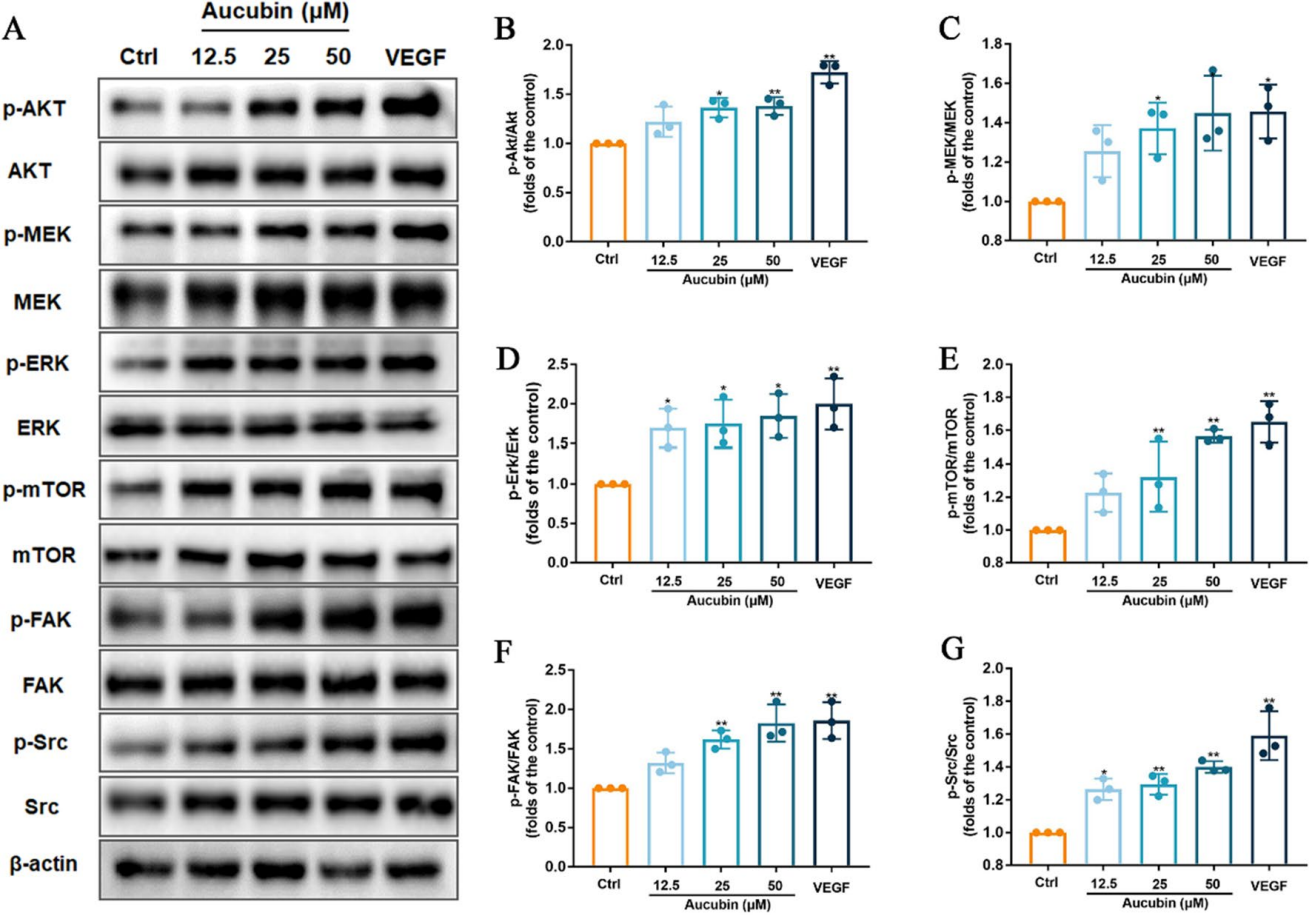
Effects of aucubin on the expression of proteins associated with the regulation of angiogenesis. HUVECs were starved in low serum media (0.5% FBS) for 3 h, then treated with VEGF (50 ng/mL, positive control) and different concentrations of aucubin (0–50 µM) for 2 h. **A** Several key proteins associated with signaling pathways of proliferation and survival in angiogenesis were determined by Western blotting. **B**–**G** Expression levels of p-Akt, p-MEK1/2, p-ERK1/2, p-mTOR, p-FAK and p-Src, respectively. Data are presented as the percentage of the control group (mean ± SD of three independent trials). *p < 0.05 and **p < 0.01 versus the control group

To explore the potential molecular mechanisms underlying the pro-angiogenic effects of aucubin, the expression levels of several key proteins (Akt, FAK, mTOR, Src, MEK and ERK1/2) associated with the regulation of proliferation and survival in angiogenesis were determined. In Fig. 5, aucubin at a concentration higher than 25 µM, increased the phosphorylation of MEK, mTOR and FAK protein. In addition, the results indicated that 12.5, 25 and 50 µM aucubin increased the protein expression levels of p-Akt, p-ERK1/2 and p-Src.

### Effects of aucubin on proliferation, migration and tube formation in su5416-injured HUVECs

**Fig. 6:**
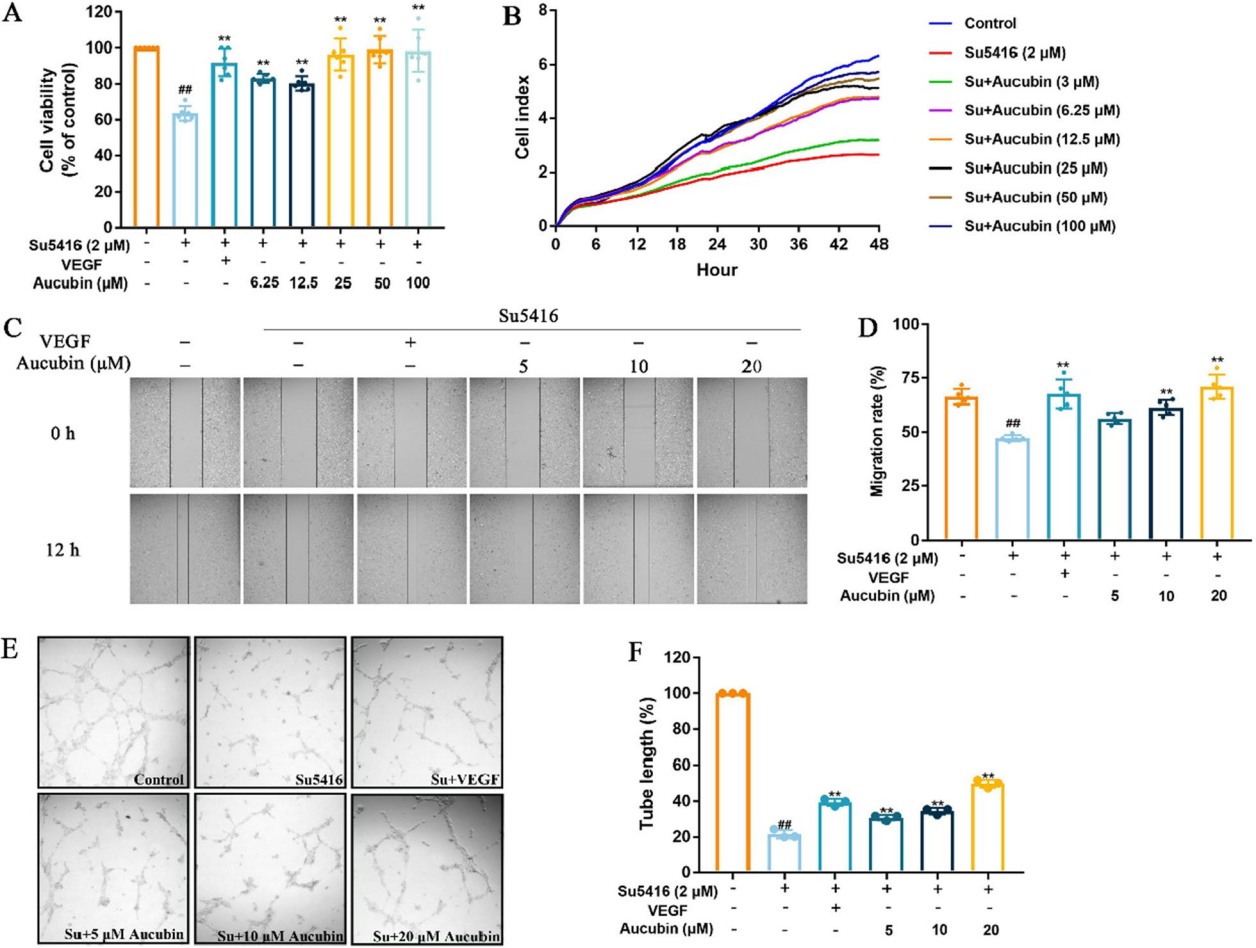
Aucubin promoted proliferation, migration and tube formation in su5416-injured HUVECs. **A** Cell viability was measured by CCK8 assays. **B** The xCELLigence RTCA system showed the data of cell index curves of HUVECs reflected their proliferation in real-time mode. **C** The healing area of the wound at the time points of 0 h and 12 h were observed microscopically and calculated by Image J software. Effects of aucubin on HUVECs migration was investigated by wound healing. **D** The migration rate was determined after treatment. **E** Effects of aucubin on tube formation in su5416-damaged HUVECs was determined. **F** Graph showing the quantitative analysis of tube length in the control group, su5416 group, and aucubin groups. Data are shown as mean ± SD of at least three independent experiments. ^##^P < 0.01 vs. untreated control. *P < 0.05 and **P < 0.01 vs. su5416

The cell viability was evaluated via the CCK8 assay to investigate the effect of aucubin on su5416-injured HUVECs proliferation. As shown in Fig. 6A, cell viability in the su5416 group decreased from 100% to 63.61 ± 4.17% compared with the control group, while 6.25–100 μM aucubin significantly increased the viability of su5416-injured HUVECs to 82.87 ± 2.58%, 81.11 ± 4.15%, 96.47 ± 8.76%, 99.13 ± 7.40% and 98.5 ± 11.52% in a concentration-dependent manner, respectively. Moreover, cell proliferation was observed for 48 h by xCELLigence Real-time cell analysis system. The cell index, reflecting cell numbers, started to decline compared to control after 6 h incubation with su5416. However, aucubin treatment could reverse the injury by su5416. The effect of aucubin on migration was evaluated by wound healing assays in su5416-injured HUVECs (Fig. 6C and D). The results showed that the migration rate was 66.45 ± 3.47% in the control group. After su5416 treatment (2 μM) for 12 h, the migration rate decreased to 47.32 ± 1.67%, causing the decelerated recovery of scratch area. After aucubin treatment for 12 h, the migratory ability significantly increased in su5416-injured HUVECs. Migration rates at 5, 10, and 20 μM were 56.69 ± 1.97%, 61.73 ± 2.56%, and 70.89 ± 5.64%, respectively. Migration of ECs is another critical step in angiogenesis, which is to form new capillary tube derived from preexisting vessels and migrated ECs. Herein, we also investigated the effect of aucubin on tube formation in su5416-injured HUVECs (Fig. 6E and F). A network of capillary-like structures was formed on the surface of Matrigel after 6 h in the control group. In su5416 group, tube length decreased from 100% to 21.54 ± 2.41% in response to su5416 compared with control. However, 5, 10, and 20 μM aucubin promoted the formation of chord-like networks in su5416-injured HUVECs, increasing tube length to 30.81 ± 1.49%, 34.41 ± 1.81%, and 49.82 ± 2.18%, respectively.

### Aucubin promoted angiogenesis by activating the VEGFR2/MEK/ERK signaling pathway in su5416-injured HUVECs

**Fig. 7:**
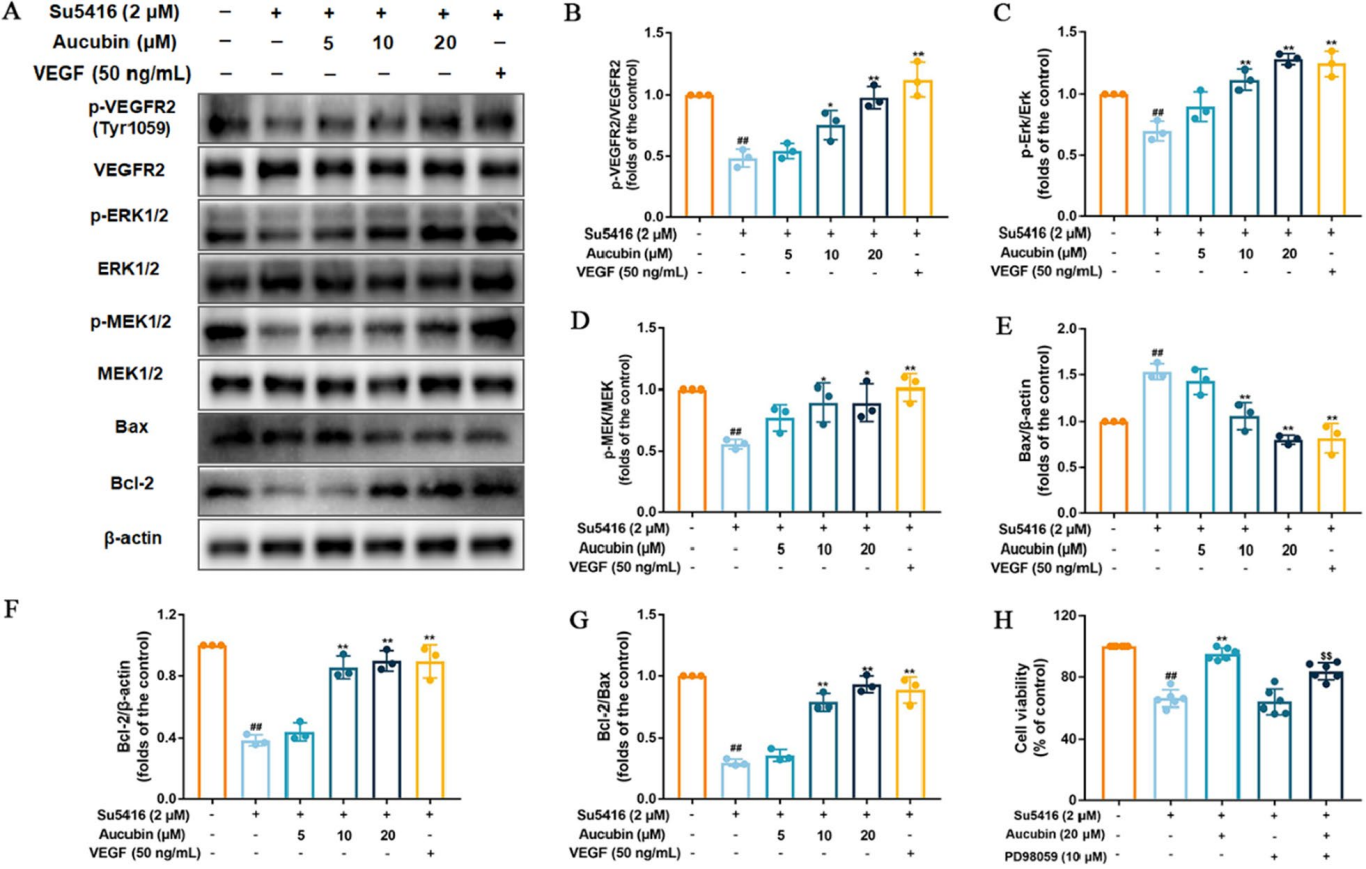
Aucubin promoted angiogenesis by regulating the VEGFR2/MEK/ERK signaling pathway. **A** The protein expression levels of p-ERK1/2, p-MEK1/2, p-VEGFR2, Bax and Bcl-2 in su5416-injured HUVECs after aucubin treatment (5, 10, and 20 μM) for 48 h were investigated by western blot. **B**–**D** Quantitative analysis of protein expression ratios of p-VEGFR2/VEGFR2, p-MEK1/2/MEK1/2 and p-ERK1/2/ERK1/2, respectively. Quantitative analysis of protein levels of Bcl-2 (**E**), Bax (**F**) and Bcl-2/Bax expression ratio (**G**) in HUVECs. (H) Cells were treated with PD98059. MEK inhibitor reversed the protective actions of aucubin on cell viability. Data are shown as mean ± SD of three independent experiments. ^#^P < 0.05 and ^##^P < 0.01 vs. untreated control. *P < 0.05 and **P < 0.01 vs. su5416. ^$$^P < 0.01 vs. su5416 + aucubin group

Encouraged by the effect of aucubin on promoting the proliferation, migration and tube formation of su5416-injured HUVECs, we next explored the potential molecular mechanisms by investigating the effect of aucubin on the VEGFR2/MEK/ERK pathway in su5416-injured HUVECs (Fig. 7). Our results demonstrated that the protein expression ratios of p-VEGFR2/VEGFR2, p-MEK1/2/MEK1/2, and p-ERK1/2/ERK1/2 were decreased in the su5416 model group after 48 h treatment compared with the control group, whereas 20 μM aucubin significantly increased the expression ratios of these proteins in su5416-injured HUVECs. The binding potential of aucubin with VEGFR2 and VEGFa-VEGFR2 complex was demonstrated using molecular docking (Additional file 1: Fig. S1). In addition, the protein expression levels of Bcl-2 and Bax were assessed. After treatment with su5416 for 48 h, the expression level of Bax was significantly increased and that of Bcl-2 was reduced (Fig. 7). However, aucubin increased Bcl-2 expression level at 5, 10 μM and 20 μM and decreased the level of Bax at 10 μM and 20 μM compared with the su5416 group. 10 and 20 μM aucubin increased the expression ratio of Bcl-2/Bax in su5416-injured HUVECs. Additionally, PD98059, a highly selective MEK/ERK inhibitor, was used to confirm whether drug exerts effects through the MEK/ERK signal pathway. We found that the cell viability in the triple-treatment group (aucubin + su5416 + PD98059) was significantly decreased in comparison with su5416 plus aucubin group (Fig. 7H). The result demonstrated that blocking the MEK activity decreased the effect of aucubin on reversing su5416-induced cell viability reduction. These results suggested that aucubin promoted angiogenesis by activating the VEGFR2/MEK/ERK signaling pathway in HUVECs.

## Discussion

Vascular neovascularization is not only a prerequisite for tissue development and regeneration, but also an important component in the pathogenesis of chronic diseases [30]. Angiogenesis plays an important role in bone fracture repair and regeneration, which contributes to pathological bone disorders such as osteoporosis, bone metastasis, and osteonecrosis [4]. In our study, aucubin could reverse the resorption of bone matrix and the loss of neural arches in RANKL-induced osteoporosis transgenic medaka by live imaging. Meanwhile, aucubin increased angiogenesis in VRI-induced vascular insufficiency zebrafish larvae and in HUVECs. There is growing descriptive evidence that various bioactive molecules from Chinese medicine simultaneously promote bone regeneration and angiogenesis to improve disease progression in animal models. Total flavonoids of *Drynariae* showed effects against osteoporosis and bone repair, as well as promoted angiogenesis-osteogenesis coupling through activating platelet-derived growth factor receptor-β signaling axis to promote type-H vessel angiogenesis in animal models [31]. Naringin, a flavonoid glycoside, has been reported to promote fracture healing in osteoporosis rats due to its pro-angiogenic activity by regulating the VEGF/VEGFR-2 signaling pathway [32]. Icariin promoted the expression of VEGF and angiopoietin 1 (ANG1) mRNA in an osteoporosis model [33]. Vitexin promotes angiogenesis and osteogenesis via the VDR/PI3K/AKT/eNOS signaling pathway in ovariectomy-induced osteoporosis of rats [34].

*Eucommia ulmoides*, which exhibited the functions of nourishing liver and kidney, strengthening muscles and bones recorded in ancient books of Chinese medicine, has been used for the treatment of osteoporosis for a long time in China [9]. Aucubin, as the major active ingredient of *Eucommia*, has been considered a potential agent for the treatment of osteoporosis due to its ability to promote bone formation and inhibit osteoclast activity [11, 35, 36]. A previous study showed that aucubin inhibit multinucleated osteoclast maturation and promote the formation of vessels in ovariectomy mice [12]. Our studies further revealed the anti-osteoporosis and pro-angiogenic effect of aucubin by live imaging in transgenic medaka and zebrafish models, respectively. A double transgenic col10α1:nlGFP/rankl:HSE:CFP medaka was heat shocked to induce the formation of ectopic osteoclasts, thereby reducing the notochordal sheath and bone mineralization. However, heat-shocked medaka were treated with 25 and 50 μM aucubin for 5 days, starting from the day of RANKL induction, the resorption of the mineralized bone matrix and centra degradation were decreased. In the skeletal system, vasculature of bone regulates skeletal development and regenerative bone formation during the embryonic stage, postnatal growth, and bone remodeling [3]. Transgenic fli1a:EGFP zebrafish that express enhanced green fluorescent protein under control of the fli1 promoter in all blood vessels has been increasingly applied as a reliable model for observing the assessment of blood vessel formation in real time visually [37]. In the present study, we found that aucubin could reverse VRI-induced vascular insufficiency in Tg(fli1a:EGFP)^y1^ zebrafish. Moreover, real-time PCR data demonstrated that aucubin exerted pro-angiogenic actions possibly via up-regulation of both VEGF-VEGFR (flt1, kdr, kdrl and vegfaa) and Ang-Tie (ang-1, ang-2, tie1 and tie2) signaling pathways in zebrafish.

The growth of new blood vessels, angiogenesis, is an essential but highly regulated multiscale process involving in complex signalling pathways. The first and the most important step towards angiogenesis is to initiate new EC proliferation [38]. Our cell proliferation results demonstrated that the concentration of 12.5, 25, and 50 μM aucubin promoted proliferation of HUVECs. These results were also confirmed by observing cell index for 48 h by xCELLigence Real-time cell analysis system, and indicated that aucubin could promote cell growth rapidly to a stage of equilibrium without induce excessive cell proliferation. The Akt/mTOR, MAPK/ERK and Src-FAK signalling pathways play a significant role in regulating cell viability and proliferation in endothelial cells [39–41]. In this study, aucubin increased the phosphorylation of Akt, mTOR, MEK, ERK1/2, FAK and Src in endothelial cells. Taken the results together, it suggested that the pro-angiogenic effects of aucubin may be regulated via the activation of Akt/mTOR, MAPK/ERK and Src-FAK signaling pathway.

VEGF receptor 2 is the primary VEGF receptor mediating proliferation, migration, and tube formation in ECs [42]. Su5416 is a selective inhibitor of the tyrosine kinase activity of the vascular endothelial growth factor (VEGF) receptor 2 [43, 44]. Therefore, this study investigated the pro-angiogenic effect and the underlying mechanisms of aucubin in su5416-damaged HUVECs. The results of cell proliferation demonstrated that 5, 10 and 20 μM aucubin promoted proliferation of su5416-damaged HUVECs and increase cell numbers observed by xCELLigence Real-time cell analysis system. New ECs migrate into the extracellular matrix to form new vascular networks and provide structural support to regenerate tissues. In this study, 10 and 20 μM aucubin obviously increased su5416-inhibited migration and tube formation in HUVECs. Taken these experimental results together, it could be concluded that aucubin exhibits pro-angiogenic activity by promoting cell proliferation, migration, and tube formation in su5416-injured HUVECs. In the pathological process of osteoporosis, blood vessels were injury and the decrease of angiogenesis leads to osteoporosis [8]. However, it has been known that abnormal angiogenesis or uncontrolled growth of blood vessels are hallmarks of many malignant diseases, especially cancer [45]. In our study, we found that aucubin showed slightly effect on promoting cell growth of normal HUVECs compared to VEGF without inducing abnormal proliferation. However, aucubin showed more pronounced potential on repairing damaged HUVECs, which indicated the promising role of aucubin on angiogenesis in a pathological condition.

VEGFR2 has been documented to stimulate angiogenesis in ECs through activating downstream pathways. Activation of MAPK family members such as MEK1/2 and ERK1/2 have been implicated to play a critical role in VEGFR2-stimulated angiogenesis by mediating the proliferation, migration, and tube formation of endothelial cell [39]. Our data showed that aucubin increased phosphorylation of VEGFR2, MEK1/2, and ERK1/2 and the expression ratio of Bcl-2/Bax in su5416-injured HUVECs. Our molecular docking results demonstrated the binding potential of aucubin with VEGFR2. It has been reported that drugs binding to the active site of VEGFa-VEGFR2 complex can increase the binding of VEGFa with VEGFR2. The enhanced binding induces conformational changes in VEGFR2, resulting in its dimerization and autophosphorylation to p-VEGFR2 [46]. Aucubin bound to the active site of VEGFa-VEGFR2 in the form of a “chain”, further implying aucubin exerts its pro-angiogenic activity probably via interactions with VEGFR2 and VEGFa-VEGFR2 complex. Additionally, PD98059 is widely used to inhibit MEK1 activation and the MAP kinase cascade. In this study, the increased cell viability of su5416-damaged HUVECs caused by aucubin was significantly reversed by the inhibitor PD98059. All the results indicated that aucubin promotes angiogenesis in su5416-injured HUVECs via regulating VEGFR2/MEK/ERK pathway.

**Fig. 8:**
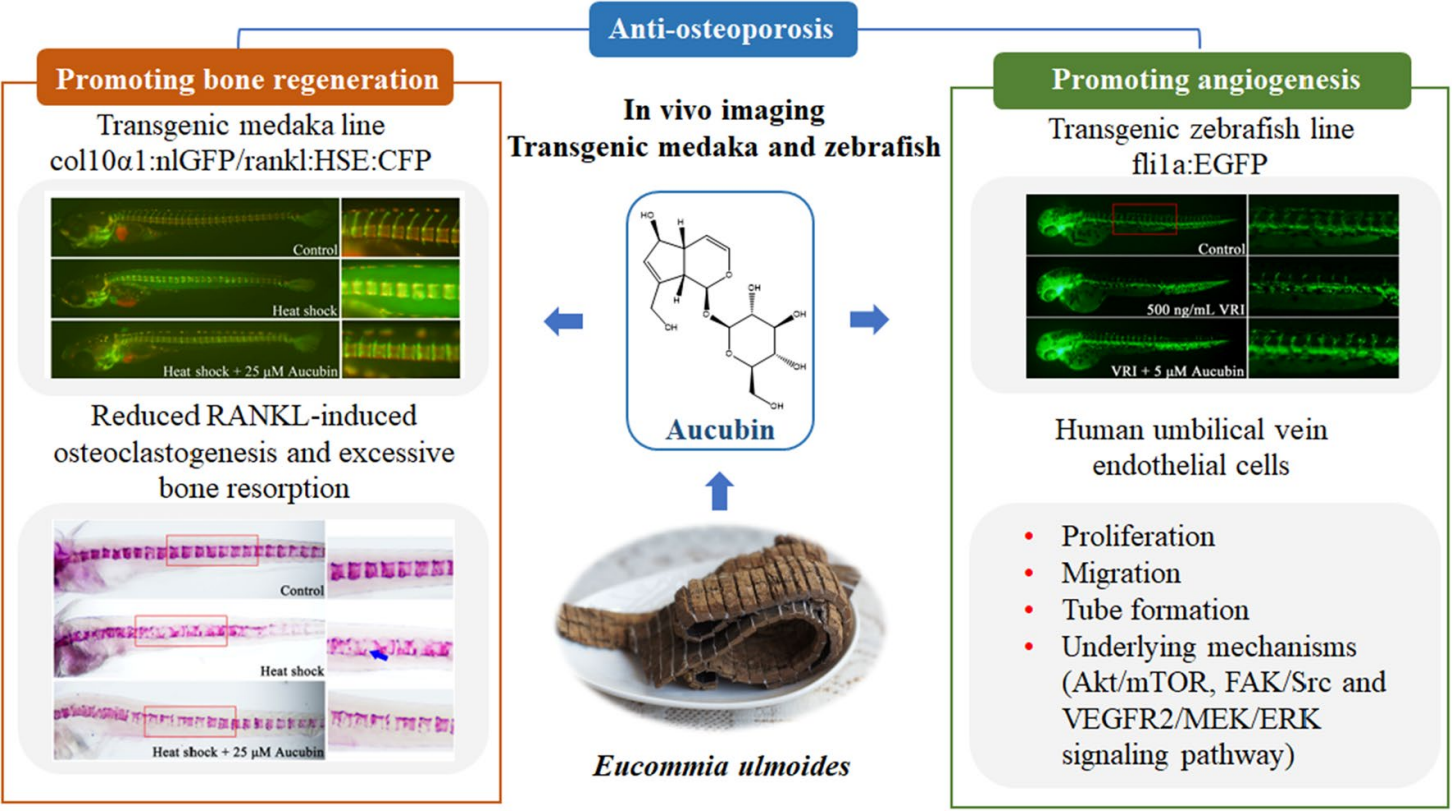
Scheme summarizing the dual effect of aucubin on promoting VEGFR2 mediated angiogenesis and reducing RANKL-induced bone resorption which may be beneficial to its treatment of osteoporosis

In previous study, aucubin slows the development of osteoporosis and promote vessel formation in osteoporosis animal models [11]. The current study further indicated that aucubin could reduce the resorption of the mineralized bone matrix and increase bone integrity of the neural arches in RANKL-induced osteoporosis medaka by live imaging. Meanwhile, aucubin increased angiogenesis through regulating VEGF-VEGFR and Ang-Tie signaling pathways in VRI-induced vascular insufficiency zebrafish larvae. The study further confirmed that aucubin promoted angiogenesis by promoting proliferation, migration, and tube formation in HUVECs and the underlying mechanisms might correlate with regulation of the Akt/mTOR, FAK/Src and VEGFR2/MEK/ERK signaling pathway. Taken together, this study further provide evidence that the effect of aucubin in the treatment of osteoporosis may due to its significant dual effects of reducing bone resorption and increasing angiogenesis (Fig. 8).

### Supplementary Information

**Additional file. **Molecular docking studies.

## Data Availability

Yes.

## Abbreviations

VEGF: Vascular endothelial growth factor
VRI: Vascular endothelial growth factor tyrosine kinase inhibitor II
HUVECs: Human umbilical vein endothelial cells
ECs: Endothelial cells
EGFP: Enhanced green fluorescent protein
F-12K: Kaighn's modification of Han's F12 medium
PS: Penicillin–streptomycin
FBS: Fetal bovine serum
PBS: Phosphate-buffered saline
EDTA: Ethylenediaminetetraacetic acid
DMSO: Dimethyl sulfoxide
PFA: Paraformaldehyde
CFP: Cyano-fluorescent protein
hpf: Hour post fertilization
RTCA: Real-time cell analyzer
ALC: Alizarin complexone
AR: Alizarin red
ISVs: Intersegmental blood vessels
ANG1: Angiopoietin 1
