# Supplementary material for: Dual effect of aucubin on promoting VEGFR2 mediated angiogenesis and reducing RANKL-induced bone resorption
Source: Chin Med. 2023 Aug 29;18:108. doi: 10.1186/s13020-023-00786-w (PMC10464038; doi:10.1186/s13020-023-00786-w)
Supplement: Supplementary file 1 — Additional file. Molecular docking studies. [file 13020_2023_786_MOESM1_ESM.docx]

*Supplementary Materials for*

**Dual effect of aucubin on promoting VEGFR2 mediated angiogenesis and reducing RANKL-induced bone resorption**

Yulin He ^1^, Hiotong Kam ^1^, Xue Wu ^1^, Qian Chen ^1, 3^, Simon Ming Yuen Lee ^1, 2, *^

^1^ *State Key Laboratory of Quality Research in Chinese Medicine and Institute of Chinese Medical Sciences, University of Macau, Avenida da Universidade, Taipa, Macao, 999078, China*

^2^ *Department of Pharmaceutical Sciences, Faculty of Health Sciences, University of Macau, Avenida da Universidade, Taipa, Macao, 999078, China*

*^3^ Center for Evolution and Conservation Biology, Southern Marine Science and Engineering Guangdong Laboratory (Guangzhou), Guangzhou, 511458, China*

**Correspondence: simonlee@um.edu.mo*

**Method**

*Molecular docking studies*

The crystal structures of VEGFR2 (PDB ID: 1vr2) [1] and VEGFa-VEGFR2 complex (3v2a) [2] were downloaded from Protein Data Bank (<https://www.rcsb.org/>). The 3D molecular structure of aucubin (Pubchem CID: 91458) was obtained from PubChem (https://pubchem.ncbi.nlm.nih.gov/). AutoDock Vina (v1.2.3) was used to perform molecular docking, and the evaluation criteria of binding degree referred to the previous study [3]. After docking, the best binding pose was visualized and analyzed by PyMOL (v2.3.0).

**Result**

An *in silico* molecular docking experiment was performed to explore the possible binding between aucubin and VEGFR2 protein. The residues involved in the hydrogen-bond interaction include Cys-919 and ASP-1046 were shown in Fig. S1A. The absolute value of binding affinity (-6.70 kcal/mol) represented the certain binding capacity of aucubin and VEGFR2. Moreover, molecular docking was also performed to assess the binding mode of aucubin with VEGFa-VEGFR2 complex. The docking score of aucubin on the VEGFa-VEGFR2 complex was -5.68 kcal/mol, which indicated suitable interactions. The binding model revealed that aucubin linked VEGFa and VEGFR2 and bound to the active site of VEGFa-VEGFR2 in the form of a “chain”, which formed one key hydrogen bond with VEGFa (Asp34) and two hydrogen bonds with VEGFR2 (Lys286 and ASP-276).


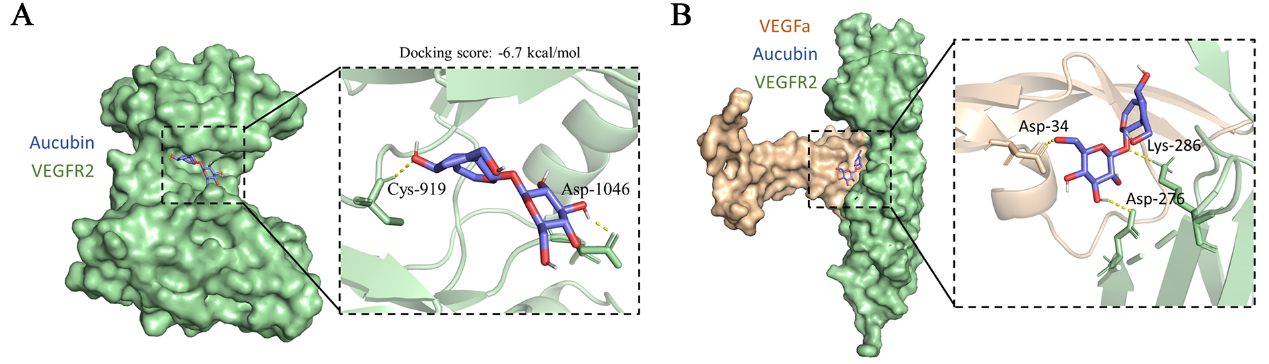


Fig. S1 The interaction of aucubin with VEGFR2 protein and VEGFa-VEGFR2 complex in molecular docking analysis. (A) Docking complex of aucubin with VEGFR2 (docking score: -6.7 kcal/mol). (B) Molecular docking of aucubin binding into the active site of VEGFa-VEGFR2 complex. Aucubin forms one key hydrogen bond with VEGFa and two hydrogen bonds with VEGFR2.

**Reference**

1. Cui H, Yang X, Wang Z, Li G, Li L, Huo S, Zhang B, He R, Chen K, Xu B, et al. Tetrahydropalmatine triggers angiogenesis via regulation of arginine biosynthesis. Pharmacol Res. 2021; 163:105242.

2. Zhong W, Yang W, Qin Y, Gu W, Xue Y, Tang Y, Xu H, Wang H, Zhang C, Wang C, et al. 6-Gingerol stabilized the p-VEGFR2/VE-cadherin/beta-catenin/actin complex promotes microvessel normalization and suppresses tumor progression. J Exp Clin Cancer Res. 2019; 38:285.

3. Wang Y, Fan Z, Yang M, Wang Y, Cao J, Khan A, Liu Y, Cheng G. Protective effects of E Se tea extracts against alcoholic fatty liver disease induced by high fat/alcohol diet: In vivo biological evaluation and molecular docking study. Phytomedicine. 2022; 101:154113.
